# Temporal and spatial transcriptional regulation of phytohormone metabolism during seed development in barley (*Hordeum vulgare* L.)

**DOI:** 10.3389/fpls.2023.1242913

**Published:** 2023-09-14

**Authors:** Pham Anh Tuan, Tran-Nguyen Nguyen, Parneet K. Toora, Belay T. Ayele

**Affiliations:** Department of Plant Science, University of Manitoba, Winnipeg, MB, Canada

**Keywords:** barley, embryo, endosperm, gene expression, plant hormone, seed development

## Abstract

Plant hormones play important roles in seed development; however, transcriptional regulation of their metabolism and levels of the respective bioactive forms during barley seed development is poorly understood. To this end, this study performed a comprehensive analysis of changes in the expression patterns phytohormone metabolism genes and levels of the respective bioactive forms in the embryo and endosperm tissues. Our study showed the presence of elevated levels of abscisic acid (ABA), bioactive forms of gibberellins (GAs), jasmonate (JA) and cytokinins (CKs), auxin and salicylic acid (SA) in the endosperm and embryo tissues at early stage of seed filling (SF). The levels of all hormones in both tissues, except that of ABA, decreased to low levels during SF. In contrast, embryonic ABA level increased during SF and peaked at physiological maturity (PM) while the endospermic ABA was maintained at a similar level observed during SF. Although its level decreased high amount of ABA was still present in the embryo during post-PM. We detected low levels of ABA in the endosperm and all the other hormones in both tissues during post-PM phase except the relatively higher levels of jasmonoyl-isoleucine and SA detected at late stage of post-PM. Our data also showed that spatiotemporal changes in the levels of plant hormones during barley seed development are mediated by the expression of specific genes involved in their respective metabolic pathways. These results indicate that seed development in barley is mediated by spatiotemporal modulation in the metabolism and levels of plant hormones.

## 1 Introduction

Seed development is a complex physiological process that determines seed yield and quality traits. Seeds of many filed crops serve as reproductive units as well as source of food, feed and raw materials for many industrial products. In cereal crops such as barley (*Hordeum vulgare*), seed development can be divided into three phases (Sabelli and Larkins, 2009; Domínguez and Cejudo, 2014). The first phase represents double fertilization, which involves the formation of embryo and endosperm, syncytium formation and endosperm cellularization. The second phase of seed development is characterized by differentiation of proliferating cells into specialized cells, endoreduplication and deposition of storage reserves. The third phase represents seed maturation, which comprises programmed cell death (PCD), a shutdown of metabolic activity as well as induction of desiccation tolerance and dormancy. Therefore, seed developmental processes strongly influence important seed traits such as size/weight and dormancy, which refers to an adaptive trait that blocks seed germination under optimal conditions (Bewley et al., 2013; Leprince et al., 2016). Given that starch constitutes up to 75% of seed dry weight in cereals, its deposition in the endosperm during seed filling (SF) is a critical determinant of seed size/weight (Rogers and Quatrano, 1983; Evers and Millar, 2002). The induction and maintenance of dormancy during seed maturation is an important trait as the incidence of preharvest sprouting (PHS) is closely associated with the degree of dormancy manifested by the seeds. PHS, which refers to the germination of seeds on the parent plant prior to harvest, is known to cause significant yield and quality losses in cereal crops (Benech-Arnold et al., 2013). It is well established that plant hormones are involved in the regulation of many seed developmental processes including SF and dormancy induction and retention (Locascio et al., 2014). However, transcriptional regulation of the metabolism of plant hormones and therefore the level of their respective bioactive forms during barley seed development is poorly understood.

ABA plays important roles in the regulation of embryo development and SF (Chandler, 1999; Sreenivasulu et al., 2010b). Previous studies have shown that both embryo and endosperm tissues of developing barley seeds produce ABA (Sreenivasulu et al., 2006; Sreenivasulu et al., 2008; Sreenivasulu et al., 2010a), and this ABA is required for seed-filling (Sreenivasulu et al., 2010b; Chen et al., 2013). Consistently, reduction in endosperm weight and starch deposition, for example in the *shrunken endosperm genetic 8* (*seg8*) mutant of barley, is closely associated with a decrease in endospermic ABA level during the early phase of seed development (Sreenivasulu et al., 2010a). In agreement with these reports, positive correlations have been shown between ABA level and the expression patterns of key starch biosynthesis genes of barley seeds (Seiler et al., 2011). ABA level in seeds is controlled by a balance between its biosynthesis and catabolism, which are regulated mainly by the actions of 9-*cis*-epoxycarotenoid dioxygenase (NCED) and ABA 8’-hydroxylase (ABA8’OH; encoded by *CYP707A* genes), respectively (Nambara et al., 2010; Tuan et al., 2018a). GAs are also known to regulate several seed developmental processes such as embryo development, cell differentiation and SF (Sercan et al., 2022). For example, GA deficiency leads to increased rate of seed abortion or significant reduction in embryo and seed growth rate (Swain et al., 1995; Swain et al., 1997). However, the levels of GAs in seeds of cereal crops such as rice (*Oryza sativa*) is reported not to be correlated with the rate of SF (Yang et al., 2001). The level of bioactive GAs in plants is determined by the balance between their biosynthesis and inactivation. GA biosynthesis is comprised of three stages in which the final stage is divided into 13-hydroxylated and non-13-hydroxylated pathways, which produce bioactive GA_1_ and GA_4_, respectively (Hedden, 2020). The biosynthesis and inactivation of GA are regulated mainly by the actions GA 20-oxidase (GA20ox) and GA 3-oxidase (GA3ox), and GA 2-oxidase (GA2ox), respectively (Yamaguchi, 2008).

It has been shown previously that seed dormancy in cereals is regulated mainly by the balance between ABA and GA, which play antagonistic roles (Tuan et al., 2018b). An imbalance in favor of ABA is required to induce and retain dormancy while an imbalance in favor of GA is necessary to promote dormancy release and germination (Rodríguez et al., 2015; Tuan et al., 2018a; Tuan et al., 2020). Developing barley seeds have been reported to manifest the highest degree of dormancy during their PM (Sreenivasulu et al., 2006; Bewley et al., 2013). Consistent with these reports, seeds exhibit high embryonic ABA/GA ratio or a peak in ABA level around the time of their physiological maturity, and this ABA level is regulated mainly by the expression of *HvNCED2* (Benech-Arnold et al., 1999; Chono et al., 2006). Moreover, the expression levels of specific ABA biosynthesis and responsive genes in the embryo of barley seeds are reported to be induced around physiological maturity while the expression levels of key GA biosynthesis genes are maintained at low level during the same period of seed development (Sreenivasulu et al., 2006; Sreenivasulu et al., 2008).

Seed developmental processes are also regulated by other plant hormones including auxin, jasmonate (JA), cytokinins (CK) and salicylic acid (SA). The major roles of auxin during seed development include controlling determination of embryo structure and size, and development of endosperm and aleurone (Locascio et al., 2014). For example, auxin activity is reported to be necessary for initiating endosperm development and maintaining its growth during seed development (Figueiredo et al., 2015). Consistently, auxin deficiency induces abnormal endosperm proliferation or premature endosperm cellularization and formation of wrinkled seeds with reduced starch content or endosperm mass. Auxin has also been implicated in the regulation of seed dormancy (Shu et al., 2016), although its role in barley with this respect remain to be elucidated. It has been shown previously that exogenous indole acetic acid (IAA) or its precursor inhibits seed germination in wheat (*Triticum aestivum*) while this effect can be reversed by seed treatment with IAA biosynthesis inhibitor or IAA antagonists (Morris et al., 1988; Ramaih et al., 2003). Consistently, dormant seeds of wheat exhibit enhanced expression levels of IAA biosynthesis genes and higher IAA level as compared to non-dormant seeds (Liu et al., 2013). The level of IAA, the most naturally occurring auxin in plants, is controlled by the balance between its biosynthesis, which is catalyzed mainly by family of tryptophan aminotransferases (TAAs) and YUCCAs (YUCs), and its inactivation, which is regulated by members of Gretchen Hagen (GH3) IAA amidosynthetase family, dioxygenase for auxin oxidation 1 (DAO1) and IAA-Leu-resistant 1 (ILR1) (Hayashi et al., 2021).

There is evidence for the involvement of JA, which includes jasmonic acid and its derivatives, in the regulation of seed developmental processes. Modulation of jasmonic acid level in developing barley seeds has been shown to be associated with cell wall invertase-mediated sucrose cleavage, which is crucial for importing photoassimilates and thereby enhancing SF (Weschke et al., 2003; Leclere et al., 2008). Previous studies also implicate jasmonic acid as a regulator of PCD in the pericarp of developing barley seeds (Sreenivasulu et al., 2006). In agreement with this report, enhanced expression of JA-related genes have been observed in older (10-14 DAA) than younger pericarps (Pielot et al., 2015). With respect to seed dormancy, specific JA biosynthesis and signaling genes exhibit upregulation during imbibition of after-ripened/non-dormant barley seeds as compared to their dormant counterparts (Barrero et al., 2009). Consistently, JA has been shown to induce dormancy decay in seeds of other cereal species such as wheat (Jacobsen et al., 2013; Nguyen et al., 2022). Jasmonoyl-isoleucine (JA-Ile) is the most biologically active form of JA and its level in plant tissues is regulated mainly by its biosynthesis, which involves several enzymes including allene oxide synthase (AOS), allene oxide cyclase (AOC) and jasmonic acid-amido synthetase/jasmonate resistant (JAR) (Schaller and Stintzi, 2009; Wasternack and Hause, 2013).

Several studies implicate CK in the regulation of embryo/endosperm development, sink size establishment, and seed dormancy and germination (Morris et al., 1993; Kucera et al., 2005). Based on whole seed analysis, Powell et al. (2013) observed much higher level of CKs at early stage of SF (10 to 12 DAA) than any other barley seed developmental stages considered in their study. Furthermore, the endosperm of developing maize (*Zea mays*) and rice seeds have been shown to contain high amounts of CK during their early stage of development, and this CK enhances cell division in both embryo and endosperm tissues (Yang et al., 2002; Tomaz and Marina, 2010). The level of CKs in plants is controlled by the balance between its biosynthesis, which is mediated mainly by isopentenyl transferase (IPT) and lonely guy (LOG), and inactivation by the actions of CK oxidase/dehydrogenase (CKX) and CK glucosyl transferases such as zeatin O-glucosyl transferase (ZOG) (Kieber and Schaller, 2018). A previous study has shown that silencing of *CKX* in barley, which leads to lower CKX activity, is associated with increased seed weight/yield (Zalewski et al., 2010). CK is also implicated as a negative regulator of seed dormancy, for example, via repressing ABA signaling (Wang et al., 2011). Consistently, CK level is higher during the later stages of seed maturation in non-dormant than dormant wheat genotypes (Tuan et al., 2019), and dormancy release due to after-ripening is associated with upregulation of *LOG* genes (Chitnis et al., 2014).

Salicylic acid is well known for plant defense against pathogens; however, it also regulates plant developmental processes. Although some studies suggest SA as a negative regulator of seed size (Leclere et al., 2008), it is reported not to have a pronounced role in the regulation of seed size/weight under normal growing conditions (Ali and Inas Abdulsattar, 2019; Çetinbaş-Genç and Vardar, 2021; Hu et al., 2022). A previous study has also indicated that SA inhibits seed germination in barley (Xie et al., 2007), reflecting its role as a positive regulator of seed dormancy. The level of SA in plants is regulated by its biosynthesis, which involves two pathways that are mediated by an isochorismate synthase (ICS) and phenylalanine ammonia-lyase (PAL), and its inactivation, which is catalyzed mainly by SA glucosyl transferase (SGT) (Vlot et al., 2009).

Despite the critical roles of plant hormones in seed developmental processes, transcriptional regulation of their metabolism and levels during seed development is still poorly understood in barley, one of the most economically important cereal crops world-wide. To gain insights into this phenomenon, the present study performed a comprehensive analysis of changes in the levels of bioactive forms of plant hormones and expression patterns of genes involved in their respective metabolic pathways in embryo and endosperm tissues of developing barley seeds.

## 2 Materials and methods

### 2.1 Plant materials

Plants of malting barley cv. Morex, which produces seeds with low level of dormancy at harvest maturity (Rasmusson and Wilcoxson, 1979; Han et al., 1996), were grown in a growth room under a 22°C/18°C (day/night) with a 16/8 h photoperiod. Seed developmental stages were determined by designating the first extrusion of yellow anthers in the spikes as 0 days after anthesis (DAA). Three independent biological replicates of developing seeds were harvested at 10, 20, 30, 40 and 50 DAA from the middle region of the spikes (~300 seeds per ~15 spikes per ~7 plants per replicate for 10, 20 and 30 DAA samples and ~200 seeds per ~10 spikes per ~5 plants per replicate for 40 and 50 DAA samples). Embryo and endosperm (endosperm + aleurone layer + pericarp) tissues were separated from the seed samples and immediately frozen in liquid nitrogen followed by storage at -80°C until further use.

### 2.2 Target gene sequence identification

Sequences of the ABA, GA, auxin/IAA, JA, CK and SA metabolism genes of barley were identified by blast searching their homologs in Arabidopsis (*Arabidopsis thaliana*) and rice against the barley genome sequence data in Ensembl Plants (http://plants.ensembl.org/). The resulting sequences of the targeted phytohormone metabolism genes of barley were confirmed by BLAST searching the respective homologs in the NCBI database. Primers of the target genes were designed from their respective nucleotide sequences using Primer 3 software (http://bioinfo.ut.ee/primer3-0.4.0/primer3/) (**Supplementary Table 1**). Gene specificity of the primer sequences was confirmed by BLAST searching them against the NCBI GenBank database, melting curve analysis and gel electrophoresis. Primer sequences of CK metabolism (*HvIPT1*, *HvIPT2*, *HvCKX1* and *HvCKX3*) and SA biosynthesis (*HvICS*, *HvPAL1* and *HvPAL6*) genes are as reported previously (Hao et al., 2018; Gasparis et al., 2019).

### 2.3 RNA extraction and quantitative RT-PCR

Total RNA extraction from the three independent biological replicates of the respective embryo and endosperm tissues and the subsequent cDNA synthesis and qPCR assays were performed as described previously (Izydorczyk et al., 2017). The qPCR assays were carried out using 5 µl of diluted cDNA as template, 1.2 µl (5 μM) of each of the forward and reverse primers (final concentration of 300 nM), 10 µL of SsoFast Eva Green Supermix (Bio-Rad, Hercules, CA, USA) and 2.6 µl of dd water in a total reaction volume of 20 µL on CFX96 real-time system (Bio-Rad). The qPCR assay of each sample was performed in duplicates with the following thermal cycling conditions: initial denaturation and DNA polymerase activation at 95°C for 3 min followed by 40 cycles of denaturation at 95°C for the 30s, annealing at 60°C for 30s and extension at 72°C for 30s. Relative transcript levels of the target genes were determined via designating one of the samples as a calibrator using a method described before (Livak and Schmittgen, 2001). *Hvβactin* was used as a reference gene for normalization.

### 2.4 Hormone levels

Extraction of ABA, GAs, IAA, JA (JA-Ile), CKs (isopentyladenine [IPA], *trans*-zeatin [*t*Z] and dihydrozeatin [dZ]) and SA was performed from three independent biological replicates of the embryo and endosperm tissues as described previously (Lackman et al., 2011; Son et al., 2016; Izydorczyk et al., 2017). Lyophilized tissues of each sample were ground into fine powder followed by homogenization of the fine powder with 6 mL of 80% (v/v) acetonitrile containing 1% (v/v) acetic acid and internal standards of the hormones studied. Subsequent extraction and purification of the hormones were performed using Oasis cartridge columns (Waters, Milford, MA, USA) as described before (Lackman et al., 2011; Son et al., 2016). Quantitative analysis of their levels was conducted using LC-ESI-MS/MS (Agilent 1260-6430; Agilent, Santa Clara, CA, USA) as reported previously (Yoshimoto et al., 2009).

### 2.5 Statistical analysis

Statistically significant differences in gene expression and hormone levels among the seed tissues and development stages were tested using two-way ANOVA and Fisher’s least significant difference (LSD) test at *P*<0.05.

## 3 Results

### 3.1 Seed phenotypes during development

**Figure 1:**
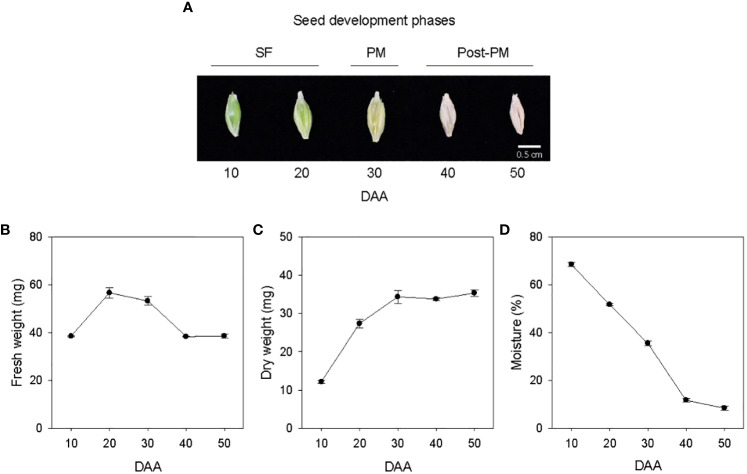
Developing seeds of barley. Malting barley cv. Morex seeds from 10 to 50 days after anthesis (DAA) **(A)**. Seed fresh weight **(B)**, dry weight **(C)** and moisture content **(D)** during seed development. Data are means ± SE (n = 30). SF, seed filling; PM, physiological maturity; post-PM; post physiological maturity.

Seed development in barley cv. Morex was studied from 10 to 50 DAA (**Figure 1A**). Both fresh and dry weights of the seeds samples increased from 10 to 20 DAA, and after 20 DAA the seed fresh weight started to decline while the dry weight continued to increase until 30 DAA but remained at a similar level thereafter (**Figures 1B, C**). Thus, the seed developmental period covered in this study was divided into three phases, SF (10-30 DAA), physiological maturity (PM, 30 DAA) and post-physiological maturity (post-PM, 40-50 DAA) (**Figure 1A**). Seeds desiccated at high rate from 10 to 40 DAA (1.6 to 2.4% moisture loss/day), and the rate of desiccation decreased markedly during transition from 40 to 50 DAA (0.34% moisture loss/day), leading to a final seed moisture content of 8.4% by 50 DAA (**Figure 1D**).

### 3.2 Expression patterns of ABA metabolism genes and ABA level

**Figure 2:**
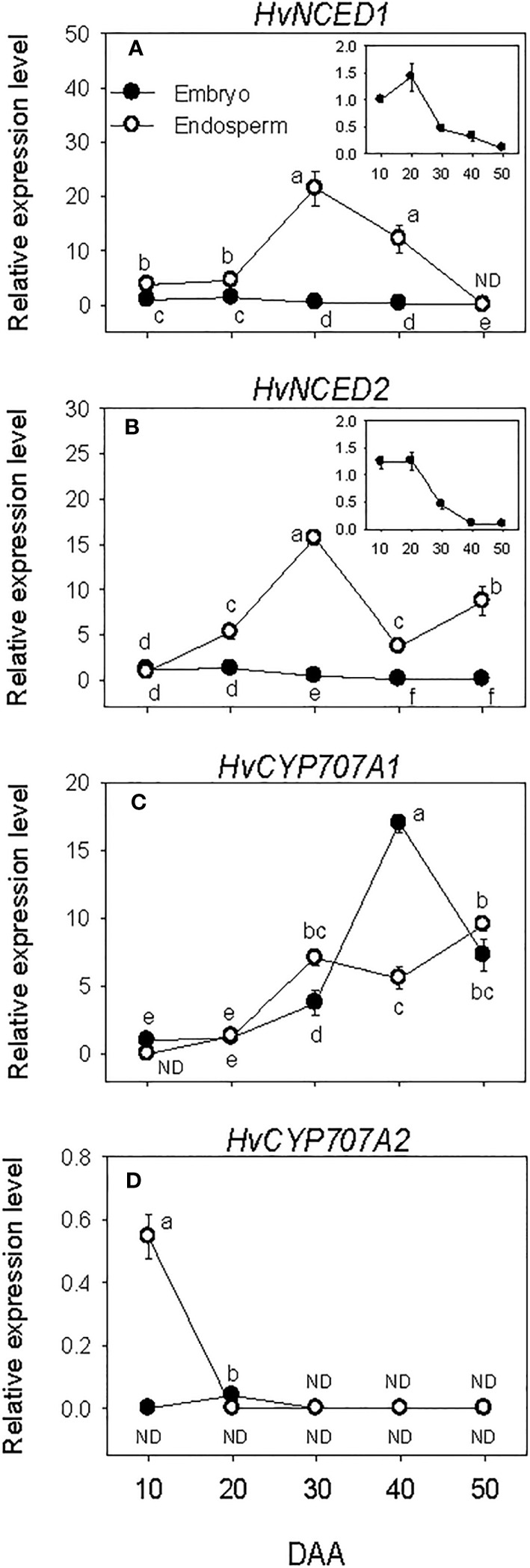
Expression of abscisic acid (ABA) metabolism genes during seed development. Relative transcript levels of *HvNCED*s **(A, B)** and *HvCYP707A*s **(C, D)** in embryo and endosperm tissues at different phases of seed development. Gene transcript levels were determined using *Taβ-actin* as reference gene, and the transcript levels of *TaNCEDs* and *TaCYP707As* were expressed relative to the transcript levels of *TaNCED1* and *TaCYP707A1* in 10 DAA embryo samples, respectively, which were arbitrarily set a value of 1. Data are means ± SE of three biological replicates. Different letters show significant difference at *P*<0.05 (LSD test). DAA, days after anthesis; ND, not detected.

Relatively higher expression levels of embryonic *HvNCED*s were observed during SF, which gradually declined to very low levels by post-PM. The expression of endospermic *HvNCED1* remained at a relatively low level during SF while that of *HvNCED2* exhibited an increase (6-fold; **Figures 2A, B**). Both genes were upregulated during transition from SF to PM phase (>3.0-fold) but downregulated during transition from PM to post-PM phase. While the expression of *HvNCED1* decreased to undetectable level during the post-PM phase, a slight increase was observed for *HvNCED2*. In general, the expression levels of *HvNCED*s were higher in the endosperm than those observed in the embryo.

Minimal to no expression of *HvCYP707A*s was detected during SF in both embryo and endosperm tissues. However, a relatively higher expression level of *HvCYP707A2* was observed in the endosperm at the early stage of SF, which decreased to undetectable level as SF progressed (**Figures 2C, D**). The expression level of *HvCYP707A1* increased (>3.2-fold) in both embryo and endosperm tissues as seeds transitioned from SF to PM phase. Its expression level in the embryo continued to increase during transition from PM to post-PM (4.5-fold) but declined (2.3-fold) during post-PM phase. In the endosperm, *HvCYP707A1* expression was maintained at a similar level during the same period. As compared to that observed in the endosperm, the expression level of *HvCYP707A1* in the embryo was slightly lower at PM (~2-fold) but higher at the early stage of the post-PM phase (3-fold). No expression of *HvCYP707A2* was detected in both embryo and endosperm tissues at PM and post-PM phases.

**Figure 3:**
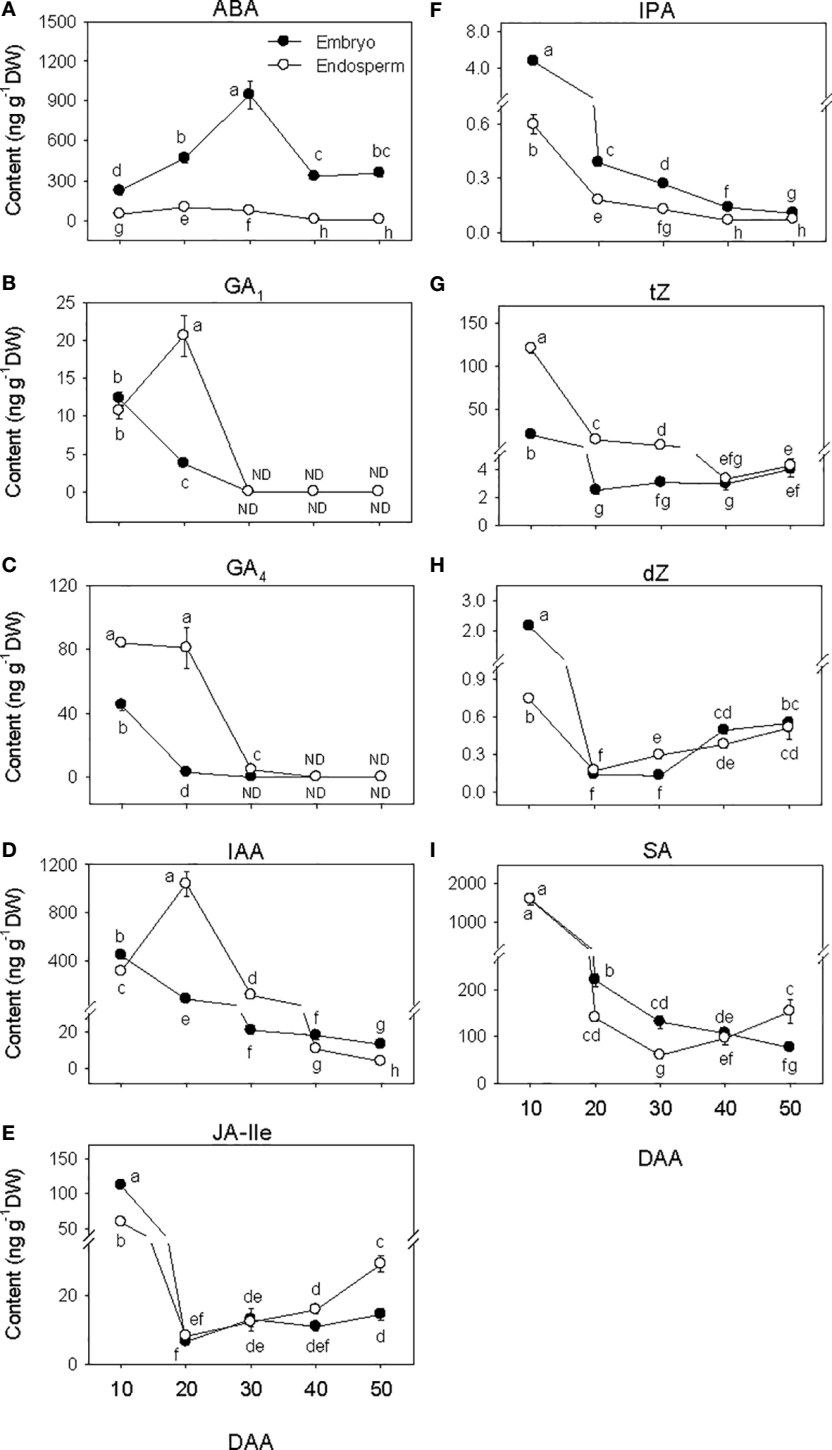
Phytohormone levels during seed development. Levels of abscisic acid (ABA, **(A)** bioactive gibberellins (GA_1_, **(B)** and GA_4_, **(C)** indole acetic acid (IAA, **D**), jasmonate [jasmonate iso-leucine (JA-Ile, **E**)], cytokinin [isopentyladenine (IPA, **F**), *trans*-zeatin (*t*Z, **G**) and dihydrozeatin (dZ, **H**)] and salicylic acid (SA, **I**) in embryo and endosperm tissues at different phases of seed development. Data are means ± SE of three biological replicates. Different letters show significant difference at *P*<0.05 (LSD test). DAA, days after anthesis; ND, not detected.

The amount of ABA in the embryo showed over 4-fold increase during SF phase (**Figure 3A**), and attained its maximum level at PM. As the seed transitioned from PM to post-PM phase, ABA level in the embryo decreased (~3-fold) and remained at a similar level thereafter. ABA was also detected in the endosperm tissue and its level increased (>2-fold) during the early stage of SF but showed a slight decline (1.3-fold) as the seed enters in to PM. Endospermic ABA level showed a drastic decline (over 9-fold) as the seed transitioned from PM to post-PM phase and remained at similar level afterward. Overall, higher level of ABA (>4-fold) was evident in the embryo than endosperm tissue during the entire duration of seed development.

### 3.3 Expression patterns of GA metabolism genes and GA levels

**Figure 4:**
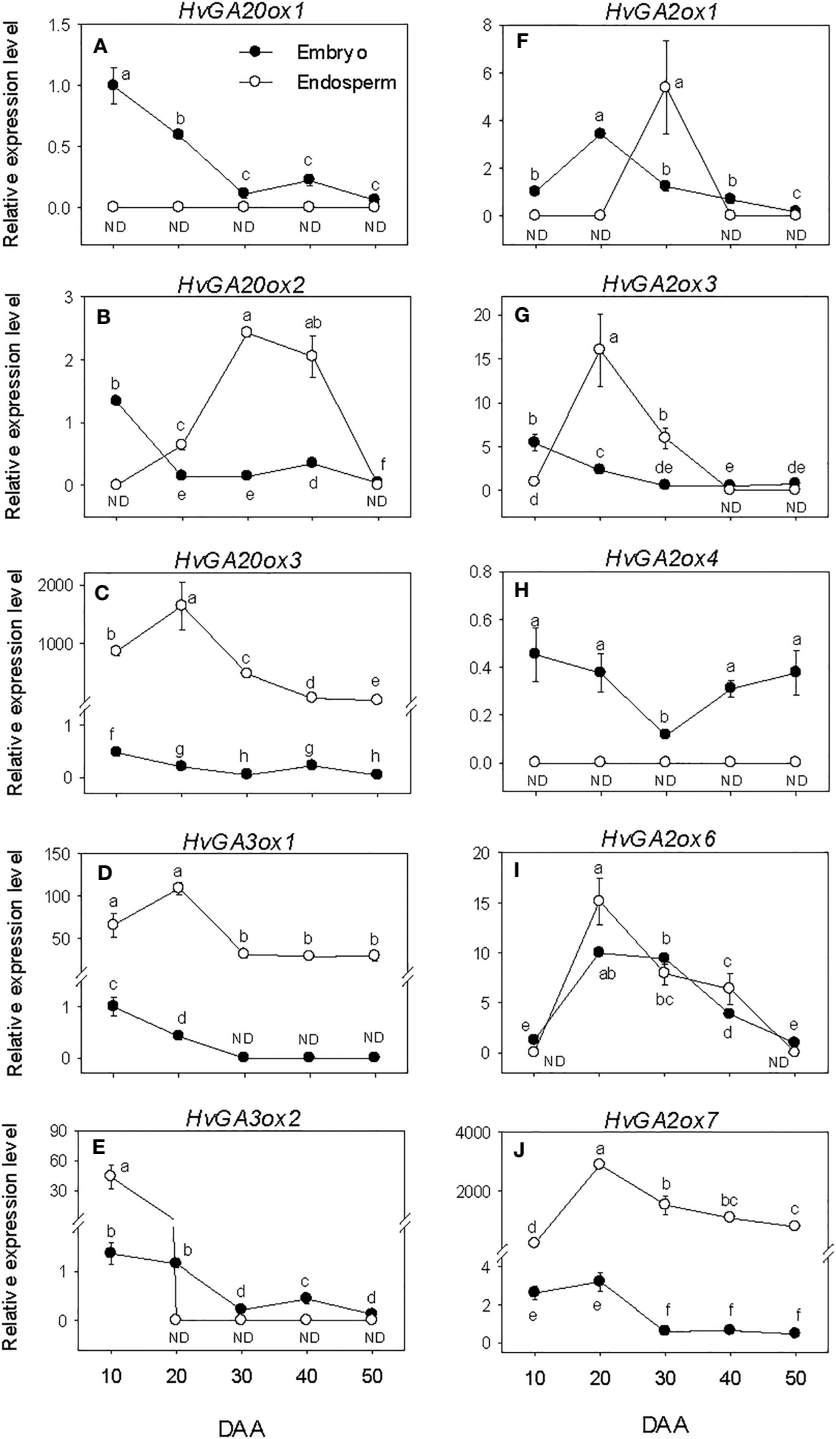
Expression of gibberellin (GA) metabolism genes during seed development. Relative transcript levels of *HvGA20ox*s **(A-C)**, *HvGA3ox*s **(D, E)** and *HvGA2ox*s **(F-J)** in embryo and endosperm tissues at different phases of seed development. Gene transcript levels were determined using *Taβ-actin* as reference gene, and the transcript levels of *HvGA20ox*s, *HvGA3ox*s, and *HvGA2ox*s were expressed relative to the transcript levels of *HvGA20ox1*, *HvGA3ox1*, and *HvGA2ox1* in 10 DAA embryo samples, respectively, which were arbitrarily set a value of 1. Data are means ± SE of three biological replicates. Different letters show significant difference at *P*<0.05 (LSD test). DAA, days after anthesis; ND, not detected.

The expression levels of embryonic GA biosynthesis genes, *HvGA20ox*s and *HvGA3ox*s decreased (over 6-fold) during SF, leading to the prevalence of low levels of their expression by PM and thereafter (**Figures 4A–E**). In the endosperm, the expression levels of *HvGA20ox3* and *HvGA3ox1* were maintained at elevated levels during the entire period of seed development. The expression level of endospermic *HvGA20ox2* exhibited an increase during SF, leading to the detection of its highest expression level at PM. A similar level of its expression detected at PM was maintained during seed transition from PM to post-PM phase before declining to undetectable level by the late stage of post-PM. No expression of endospermic *HvGA20ox1* and *HvGA3ox2* was detected during seed development except the high expression level of *HvGA3ox2* observed at the early stage of SF. Endospermic *HvGA20ox3* and *HvGA3ox1* showed much higher levels of expression than any other member of their respective gene families in both tissue. Whereas *HvGA20ox1* and *HvGA3ox2* exhibited relatively higher levels of expression in the embryo than endosperm during the entire period of seed development.

Expression levels of embryonic *HvGA2ox*s showed either maintenance at similar levels or slight decline during the entire period of seed development except that of *HvGA2ox6* exhibited an increase through PM (>3.4-fold) followed by a gradual decline afterward (**Figures 4F–J**). The expression levels of endospermic *HvGA2ox3*, *HvGA2ox6* and *HvGA2ox7* increased (>14-fold) during the earlier stage of SF but exhibited slight/gradual decrease to low levels through PM and thereafter (**Figures 4G, I , J**). No expression of endospermic *HvGA2ox1* and *HvGA2ox4* was detected at all phases of seed development except the transient increase observed for *HvGA2ox1* at PM (**Figures 4F, H**). Overall, higher expression levels of *HvGA2ox1* and *HvGA2ox4* were apparent in the embryo than endosperm during the entire period of seed development while *HvGA2ox7* exhibited higher expression level in the endosperm than embryo. Based on their relative expression levels, *HvGA2ox7* showed much higher expression level in the endosperm than any other *GA2ox* gene family member at all developmental phases. Whereas *HvGA2ox6* exhibited higher expression level in the embryo than the other family members at all developmental phases except at early stage of the SF phase and late stage of the post-PM phase.

The levels of embryonic GA_1_ and GA_4_ detected at the early stage of SF showed marked reduction (over 3-fold) as SF progressed, and no GA_1_ or GA_4_ was detected at PM and thereafter (**Figures 3B, C**). The level of endospermic GA_1_ increased (2-fold) during the early stage of SF while that of GA_4_ was maintained at similar elevated level. The levels of both GA_1_ and GA_4_ decreased to undetectable level during transition from SF to PM and remained at similar levels afterward. Higher levels of both GA_1_ and GA_4_ (over 2-fold) were observed in the endosperm as compared to that detected in the embryo during the SF phase.

### 3.4 Expression patterns of IAA metabolism genes and IAA level

**Figure 5:**
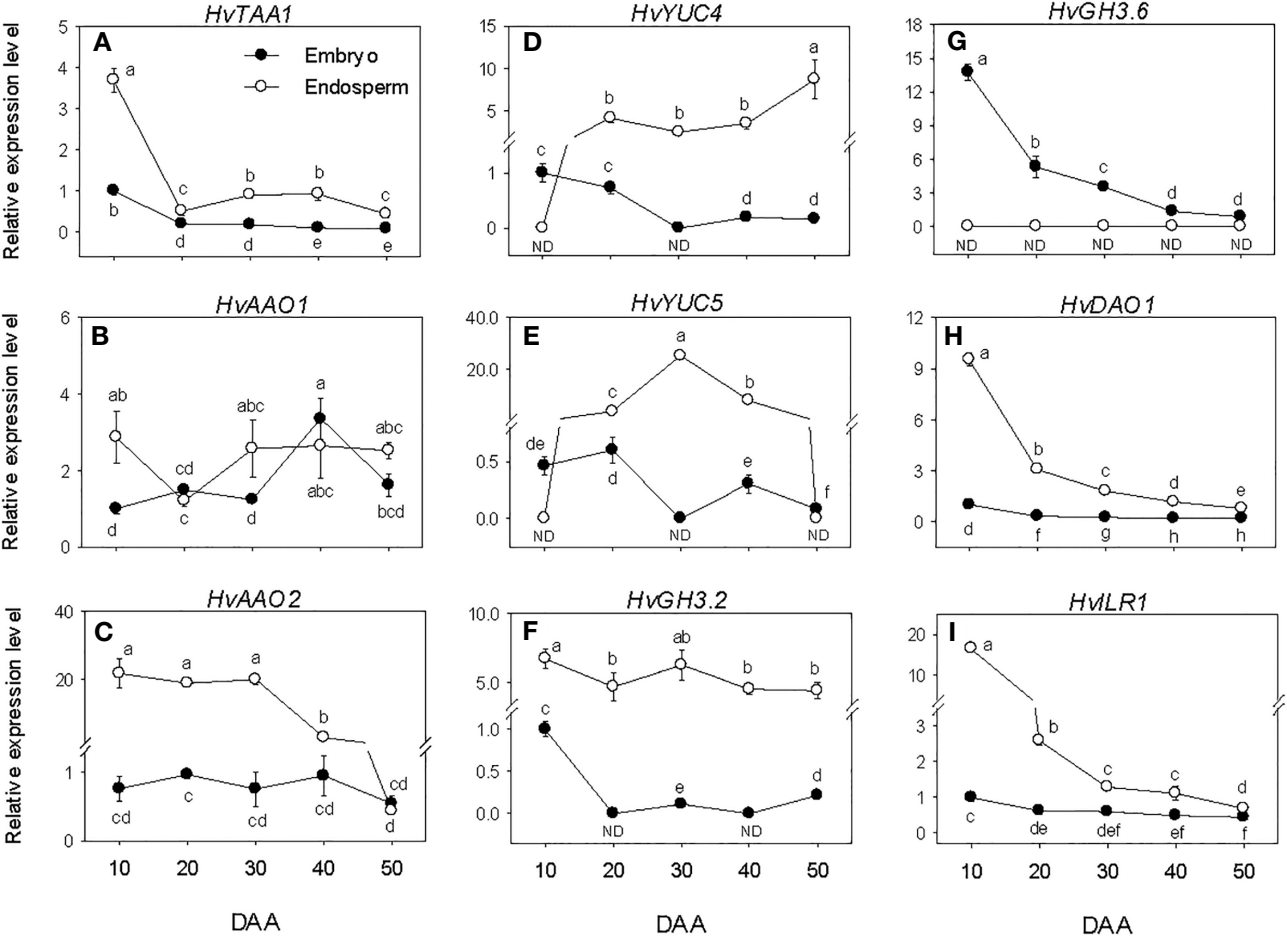
Expression of indole acetic acid (IAA) metabolism genes during seed development. Relative transcript levels of *HvTAA1* **(A)**, *HvAAO*s **(B, C)**, *HvYUC*s **(D, E)**, *HvGH3s* **(F, G)**, *HvDAO1* **(H)** and *HvILR1* **(I)** in embryo and endosperm tissues at different phases of seed development. Gene transcript levels were determined using *Taβ-actin* as reference gene, and the transcript levels of *HvTAA1*, *HvAAO*s, *HvYUC*s, *HvGH3s*, *HvDAO1* and *HvILR1* were expressed relative to the transcript levels of *HvTAA1*, *HvAAO1*, *HvYUC4*, *HvGH3.2*, *HvDAO1* and *HvILR1* in 10 DAA embryo samples, respectively, which were arbitrarily set a value of 1. Data are means ± SE of three biological replicates. Different letters show significant difference at *P*<0.05 (LSD test). DAA, days after anthesis; ND, not detected.

The expression levels of embryonic *HvTAA1, HvAAO*s and *HvYUC*s were maintained at almost similar low/undetectable levels throughout the entire seed developmental phases studied. However, a transient increase in expression level was observed for *HvAAO* at the early stage of post-PM phase (**Figures 5A–E**). The high expression level of *HvTAA1* detected in the endosperm decreased to low level during the early stage of SF (over 7-fold) and remained at almost similar level afterward. The expression level of endospermic *HvAAO1* was maintained at a similar level throughout the entire period of seed development while that of *HvAAO2* was elevated during SF and at PM but decreased gradually to very low level afterward. The expression levels of both *HvYUC4* and *HvYUC5* increased from undetectable to high level during SF, and remained at similar elevated level thereafter except that of *HvYUC5* declined to undetectable level during post-PM phase. Overall, *HvTAA1*, *HvAAO2*, *HvYUC4* and *HvYUC5* exhibited higher expression levels in the endosperm than embryo except that *HvYUC4* and *HvYUC5* showed higher expression levels in the embryo at the early stage of SF, and *HvAAO2* and *HvYUC5* exhibited similar low levels of expression in both tissues during the later stage of post-PM.

The *HvGH3.2*, *HvDAO1* and *HvILR1* genes exhibited no or minimal expression levels during the seed developmental phases studied. Relatively higher expression level of *HvGH3.6* was observed at early stage of SF, which gradually decreased to very low level as seed development progressed (**Figures 5F–I**). *HvGH3.2*, *HvDAO1* and *HvILR1* showed higher levels of expression in the endosperm than embryo during the entire period of seed development, whereas *HvGH3.6* was expressed at a higher level in the embryo than endosperm.

Embryonic IAA level exhibited a gradual decline as seed development progressed from SF through post-PM phases (**Figure 3D**). The level of IAA in the endosperm increased (over 3-fold) during the early stage of SF before exhibiting a gradual decrease thereafter. The endosperm appeared to contain a higher level of IAA (over 5-fold) during the late stage of SF and at PM while slightly higher level of IAA was apparent in the embryo at the early stage of SF and during post-PM phase.

### 3.5 Expression patterns of JA biosynthesis genes and JA-Ile level

**Figure 6:**
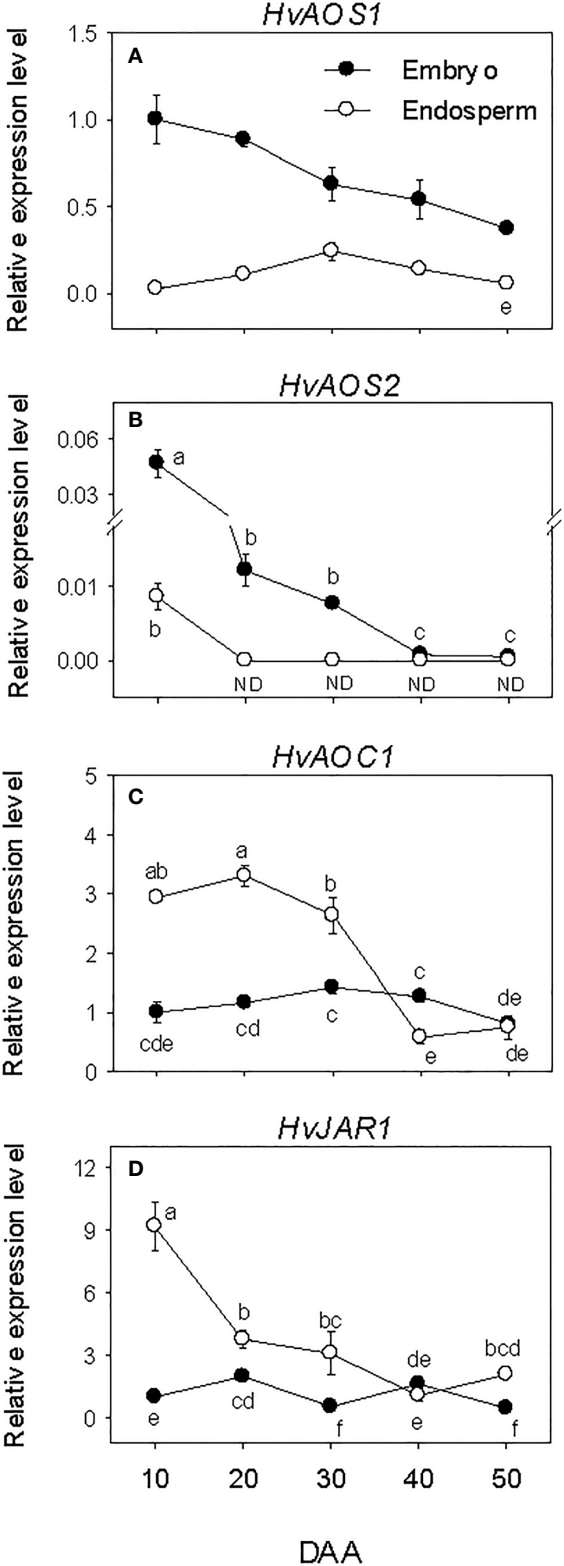
Expression of jasmonate (JA) biosynthesis genes during seed development. Relative transcript levels of *HvAOS*s **(A, B)**, *HvAOC1* **(C)** and *HvJAR1* **(D)** in embryo and endosperm tissues at different phases of seed development. Gene transcript levels were determined using *Taβ-actin* as reference gene, and the transcript levels of *HvAOS*s, *HvAOC1* and *HvJAR2* were expressed relative to the transcript levels of *HvAOS1*, *HvAOC1* and *HvJAR2* in 10 DAA embryo samples, respectively, which were arbitrarily set a value of 1. Data are mean ± SE of three biological replicates. Different letters show significant difference at *P*<0.05 (LSD test). DAA, days after anthesis; ND, not detected.

Embryonic *HvAOS*s exhibited relatively higher levels of expressions during SF, after which their expression levels declined gradually to lower levels. The expressions of *HvAOC1* and *HvJAR1* were maintained at similar low level throughout the entire period of seed development (**Figures 6A–D**). In the endosperm, minimal or no expression of the *HvAOSs* was detected at all phases of seed development. In contrast, elevated expression levels of *HvAOC1* and *HvJAR1* were observed at early stage of SF, and a similar level was maintained through PM for *HvAOC1* before declining (over 4-fold) to very low level afterward. The expression level of *HvJAR1* declined (over 2-fold) through PM and continued to decrease thereafter. Overall, higher expression levels of *HvAOS*s (>2.5-fold) were evident in the embryo than endosperm during the entire period of seed development while *HvAOC1* and *HvJAR1* exhibited higher levels of expressions (~2-fold) in the endosperm than embryo during SF and at PM (**Figures 6C, D**).

The levels of embryonic and endospermic JA-Ile decreased 17-fold and over 7-fold, respectively, during early stage of SF, and almost similar low level of JA-Ile was maintained in both tissues as the seed transitioned to PM (**Figure 3E**). The levels of embryonic and endospermic JA-Ile observed at PM were maintained during transition to post-PM and thereafter except that endospermic JA-Ile increased (~2-fold) during post-PM phase. Higher level of JA-Ile (~2-fold) was observed in the embryo at early stage of SF but its level in the endosperm was higher at late stage of post-PM phase.

### 3.6 Expression patterns of cytokinin metabolism genes and cytokinin levels

**Figure 7:**
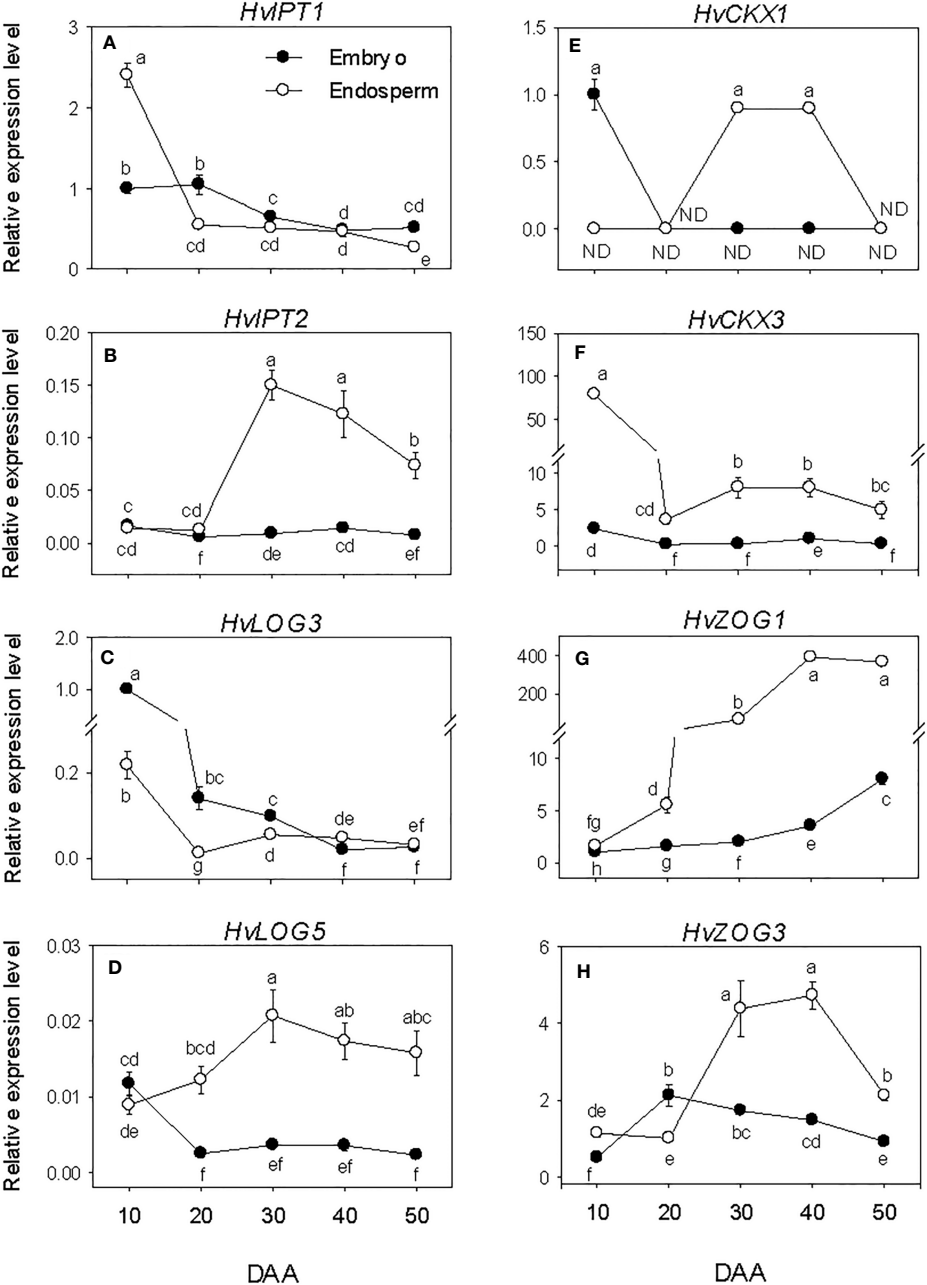
Expression of cytokinin (CK) metabolism genes during seed development. Relative transcript levels of *HvIPT*s **(A, B)**, *HvLOGs* **(C, D)**, *HvCKX*s **(E, F)** and *HvcZOG*s **(G, H)** in embryo and endosperm tissues at different phases of seed development. Gene transcript levels were determined using *Taβ-actin* as reference gene, and the transcript levels of *HvIPT*s, *HvLOGs*, *HvCKX*s and *HvcZOG*s were expressed relative to the transcript levels of *HvIPT1*, *HvLOG3*, *HvCKX1* and *HvcZOG1* in 10 DAA embryo samples, respectively, which were arbitrarily set a value of 1. Data are mean ± SE of three biological replicates. Different letters show significant difference at *P*<0.05 (LSD test). DAA, days after anthesis; ND, not detected.

All the CK biosynthesis genes except *HvIPT2* showed relatively higher expression levels in the embryo at early stage of SF after which their expressions decreased to low levels (**Figures 7A–D**). *HvIPT2* was expressed at very low level throughout the entire period of seed development. The endospermic *HvIPT1* and *HvLOG3* genes showed similar expression patterns as their embryonic counterparts. In contrast, endospermic *HvIPT2* and *HvLOG5* exhibited marked increases during early and/or late stages of SF, leading to the prevalence of their highest level of expression by PM, which was maintained through post-PM. *HvIPT2* and *HvLOG5* exhibited higher levels of expression in the endosperm than embryo during seed development except at early stage of SF. Whereas *HvLOG3* showed higher level of expression in the embryo during SF and at PM.

The embryonic *HvCKX1* and *HvCKX3* exhibited relatively higher levels of expression at early stage of SF and their expression levels decreased to either undetectable or low levels thereafter (**Figures 7E, F**). While the expression level of embryonic HvZOG1 increased gradually during seed development, that of HvZOG3 exhibited an increase during the early stage of SF followed by a very gradual decrease thereafter (**Figures 7G, H**). In the endosperm, the expression of *HvCKX1* was detected only at PM and early stage of post-PM while *HvCKX3* showed high level of expression at early stage of SF but decreased markedly (22-fold) by late stage of SF and remained at almost similar level during the subsequent phases of seed development. The expression levels of the two endospermic *HvZOG*s increased during seed development except that the expression of *HvZOG3* decreased at the late stage of post-PM. Between the two endospermic *HvCKX* genes, *HvCKX3* exhibited a much higher level of expression during seed development and its expression level in the endosperm was markedly higher than that observed in the embryo. Likewise, endospermic *HvZOG1* showed a higher level of expression than that of *HvZOG3* during seed development, and the expression levels of both genes were significantly higher in the endosperm than embryo.

The levels of all three forms of bioactive CK decreased (>3.3-fold) in both embryo and endosperm during early stage of SF after which similar levels were maintained (*t*Z) or their levels increased slightly (dZ) or decreased gradually (IPA) through post-PM phase (**Figures 3F–H**). Overall, a higher level of *t*Z was observed in the endosperm than embryo during SF and at PM while higher level of IPA was detected in the embryo than endosperm during all phases of seed development. Higher level of dZ was also prevalent in the embryo at early stage of SF.

### 3.7 Expression patterns of salicylic acid metabolism genes and salicylic acid levels

**Figure 8:**
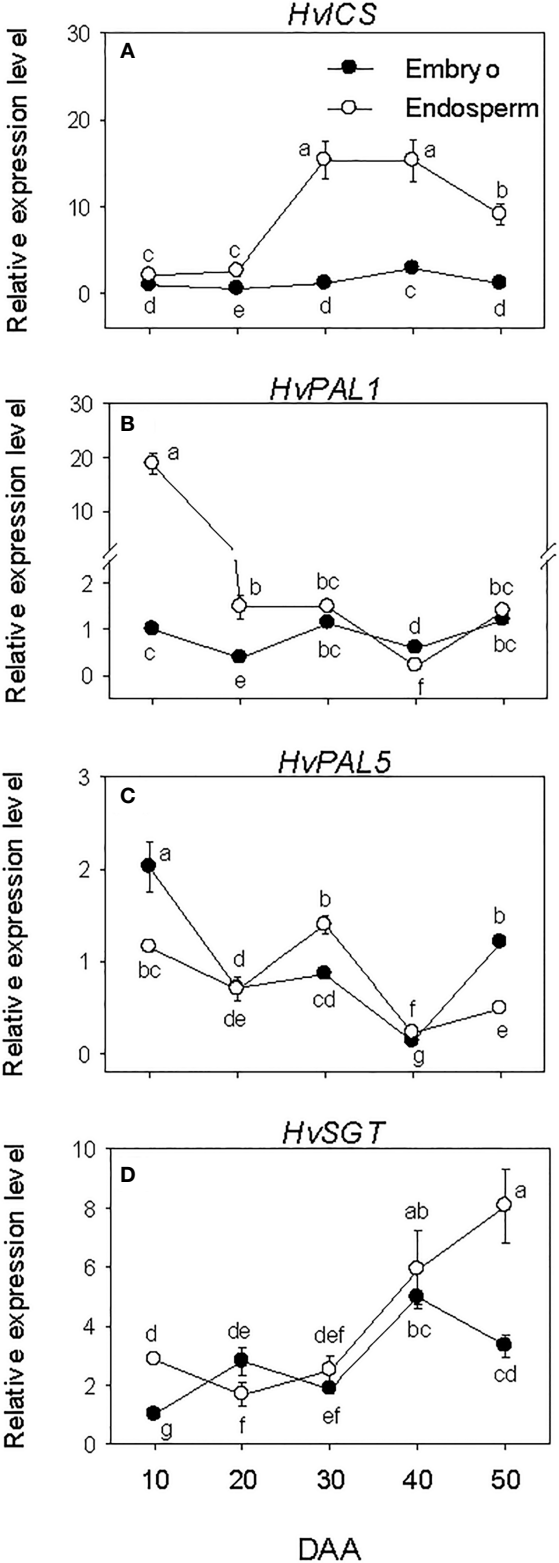
Expression of salicylic acid (SA) metabolism genes during seed development. Relative transcript levels of *HvICS* **(A)**, *HvPALs* **(B, C)** and *HvSGT* **(D)** in embryo and endosperm tissues at different phases of seed development. Gene transcript levels were determined using *Taβ-actin* as reference gene, and the transcript levels of *HvICS*, *HvPALs*, and *HvSGT* were expressed relative to the transcript levels of *HvICS*, *HvPAL1* and *HvSGT* in 10 DAA embryo samples, respectively, which were arbitrarily set a value of 1. Data are mean ± SE of three biological replicates. Different letters show significant difference at *P*<0.05 (LSD test). DAA, days after anthesis.

Embryonic *HvICS* and *HvPAL* genes expressed consistently at low levels during the entire phases of seed development except the relatively higher expression level of *HvPAL5* detected at early stage of SF (**Figures 8A–C**). In the endosperm, low expression level of *HvICS* was detected at early stage of SF followed by a marked increase (~6-fold-fold) of its expression level during transition from SF to PM and maintenance at similar level thereafter. The expression levels of *HvPAL1* and *HvPAL5*; however, showed decline (1.7- to 13-fold) during the early stage of SF, after which the expression of *HvPAL1* was maintained at a similar level while that of *HvPAL5* decreased further except the slight transient increase observed at PM. Of all the SA biosynthesis genes, *HvICS* showed consistently higher expression level in the endosperm than embryo during seed development while *HvPAL1* exhibited higher expression level in the endosperm only prior to PM. In contrast, *HvPAL5* exhibited higher expression level in the embryo at early stage of SF (1.7-fold) and late stage of post-PM (over 2-fold). The expression level of *HvSGT* increased gradually in both embryo and endosperm tissues during seed development except it decreased slightly in the embryo at late stage of post-PM (**Figure 8D**). Our data also showed higher expression level of *HvSGT* in the endosperm than embryo at the early stage of SF (~3-fold) and late stage of post-PM (over 2-fold).

The level of SA decreased (over 7-fold) in both embryo and endosperm tissues during SF and as the seed transitioned from SF to PM phase (over 1.7-fold; **Figure 3I**). While the level of SA in embryo continued to decrease during post-PM phase (1.4-fold), its level in the endosperm showed slight increase during transition from PM to post-PM phase and thereafter. As compared to that detected in the endosperm, the level of SA in the embryo was higher (over 1.6-fold) at late stage of SF as well as at PM but lower (2-fold) at late stage of post-PM phase.

## 4 Discussion

The present study investigated the spatial and temporal transcriptional regulation of phytohormone metabolism and levels during seed development in barley. Our data showed that SF in a developing barley seed peaks at 20 DAA and the seed attains a maximum dry weight, which marks its physiological maturity, by 30 DAA as reported previously (Benech-Arnold et al., 1999; Sreenivasulu et al., 2008; Seiler et al., 2011). The prevalence of an increase in ABA level in both embryo and endosperm tissues during SF reflects its importance in the regulation of embryo development and deposition of storage reserves. Consistently, ABA has been reported to induce the development of embryo and regulate endosperm weight and starch content in developing barley seeds (Chandler, 1999; Sreenivasulu et al., 2010a). The increase in endospermic ABA level observed during SF is associated with upregulation of *HvNCED*s and downregulation of *HvCYP707A2*, indicating the importance of both ABA biosynthesis and catabolism in regulating ABA level and thereby storage reserve accumulation. However, the amount of ABA in the endosperm was much lower than that observed in the embryo of developing barley seeds although the endosperm exhibited more enhanced expression levels of the two ABA biosynthesis genes. Given endospermic genes encoding proteins that act as positive regulators of ABA signaling (Chen et al., 2013) as well as those involved in sucrose hydrolysis and starch biosynthesis (Sreenivasulu et al., 2006; Sreenivasulu et al., 2008; Seiler et al., 2011) are upregulated during SF in barley, our data implicate the significance of both ABA level and sensitivity in regulating starch deposition in the endosperm. PCD in the endosperm and aleurone tissues is a major physiological event occurring during maturation of cereal seeds (Domínguez and Cejudo, 2014). Previous reports implicated ABA as a negative regulator of PCD in these tissues of barley mainly via inhibiting the synthesis of ethylene, which acts as a positive regulator of PCD (Bethke et al., 1999). Therefore, the prevalence of low ABA level in the endosperm, which also consists of the aleurone tissue in this study, during the post-PM phase might reflect its importance in facilitating programmed death of endospermic cells.

The detection of relatively high levels bioactive GAs in both embryo and endosperm tissues at the early stage of SF may implicate their contribution in controlling embryo development, differentiation of proliferating cells into specialized cells, organ expansion and SF (Sabelli and Larkins, 2009; Domínguez and Cejudo, 2014; Sercan et al., 2022). However, the levels of bioactive GAs were much higher in the endosperm than embryo during SF phase, reflecting the major role they play in regulating endosperm growth and accumulation of storage reserves. In agreement with our results, whole seed based analysis of developing seeds of barley cv. Himalaya detected higher levels of bioactive GAs during the early stage of SF (Chandler, 1999). Reduction in the levels of endospermic bioactive GAs as SF progresses is consistent with previous reports that implicated GA to have a negative effect on the rate of SF in other cereal species such as rice (Yang et al., 2001) although this phenomenon remain to be studied in barley. The observation of close associations between the decline in endospermic GA level with downregulation of *HvGA3ox2* and upregulations of *HvGA2ox*s indicates the contributions of both GA biosynthesis and catabolism in controlling bioactive GA levels during SF. Our results also showed that the difference in the levels of bioactive GAs between the endosperm and embryo tissues during SF is associated with the expression patterns specific GA biosynthesis (*HvGA20ox3*, *HvGA3ox1* and *HvGA3ox2*) and GA catabolism (*HvGA2ox1* and *HvGA2ox4*) genes. GA has also been reported to induce PCD in the aleurone of imbibing barley seeds (Domínguez and Cejudo, 2014); however, no bioactive GA was detected in the endosperm during post-PM phases. It is therefore likely that the PCD taking place in the endosperm of barley during seed maturation is not GA dependent.

Auxin play important roles in regulating embryo structure and size, and SF via controlling endosperm development and stimulating photoassimilate transport (Darussalam et al., 1998; Chandler, 1999; Locascio et al., 2014). In agreement with this report, we observed relatively higher embryonic and endospermic IAA level during SF than other developmental phases of barley seeds. However, the detection of higher level of IAA in the endosperm than embryo during the same period might suggest the major role of auxin in regulating endosperm developmental processes and accumulation of photoassimilates in barley seeds. A study that involved analysis of whole seed of barley has also shown an increase of IAA level during early stage of SF (8 to 20 DAA) (Chandler, 1999). In support of these results, auxin deficiency in developing seeds of pea (*Pisum sativum*) is associated with a decrease in the partitioning of sucrose into storage starch (Mcadam et al., 2017; Meitzel et al., 2021). The prevalence of close associations between endospermic IAA level and expression patterns of *HvAAO2*, *HvYUC4* and *HvYUC5* during SF indicates the importance of IAA biosynthesis rather than its inactivation in regulating IAA level in the endosperm. Our data also highlight that the difference in IAA level between the endosperm and embryo tissues is associated with the expression patterns of specific genes involved in its biosynthesis (*HvAAO2*, *HvYUC4* and *HvYUC5*) and inactivation (*HvGH3.6*). In general, auxin is considered as a suppressor of PCD (Kacprzyk et al., 2022); thus, the detection of very low level of endospermic IAA as the seed matures implies the occurrence of enhanced PCD in the endosperm during barley seed maturation.

Jasmonate plays a role in the regulation of embryo development and SF (Weschke et al., 2003; Leclere et al., 2008; Wasternack et al., 2013). Therefore, the observation of high level of embryonic and endospermic JA-Ile during early stage of SF in barley might indicate its role in regulating embryo developmental processes and accumulation of storage reserves. Consistently, reduction of JA-Ile level in cereal species such as rice has been shown to lead to a decrease in seed yield via decreasing the number of spikelets and SF rate (Kim et al., 2009). However, further study is required to elucidate the role of JA in regulating these phenomena in barley. The close association between the level of endospermic JA-Ile and expression pattern of *HvJAR1* during the same period of seed development might imply the importance of JA biosynthesis in regulating JA-Ile level. Previous studies have also shown the presence of high level of jasmonic acid in the pericarp of maturing barley seeds and its involvement in PCD (Sreenivasulu et al., 2006). Given the tissue considered as endosperm in this study comprises the pericarp, the prevalence of an increase in endospermic JA-Ile level during the post-PM phase might suggest that most of the JA-Ile detected in the endosperm originate from the pericarp where it regulates PCD. This hypothesis is well supported by the presence of higher level of JA-Ile in the endosperm than embryo at late stage of post-PM phase. Our study also revealed that the difference in JA-Ile level between the two tissues is mediated by the expression of *HvJAR1*.

Cytokinins play important roles in the development embryo and endosperm tissues and determination of sink size, and their levels have been shown to be associated with SF (Morris et al., 1993; Yang et al., 2000; Kong et al., 2015). Consistent with these reports, our data showed the presence of high levels of IPA, *t*Z and dZ in the embryo during early stage of SF and in the endosperm during SF. Although their analysis was based on whole seed, Powell et al. (2013) also observed much higher level of IPA, *t*Z and dZ during SF (10 to 12 DAA) than any other stage of seed development they studied. Our data also revealed the detection of higher levels of IPA and *t*Z in the embryo and endosperm, respectively, during SF and at PM of developing barley seeds, indicating the major bioactive forms of CK produced in barley seeds vary with tissue and stage of development. The levels of endospermic CKs during SF is closely associated with the expression patterns of *HvIPT1* and *HvLOG3*, and *HvZOG1* and *HvZOG3*, reflecting the importance of both CK biosynthesis and inactivation in controlling the levels of CKs and thereby endosperm development and accumulation of storage reserves. It appears from our data that the difference in IPA level between the endosperm and embryo tissues during SF and at PM is mediated mainly by the expression of *HvLOG3* while the difference in *t*Z level between the two tissues is regulated by the expression of *HvIPT1* and *HvIPT2* and/or *HvLOG5*. Although CK induces PCD (Vescovi et al., 2012), the observation of low levels of CKs during post-PM phase of barley seeds imply that its role in regulating barley seed developmental processes is restricted mainly to the early stage of SF, and other seed maturation associated events such as PCD are CK independent.

The plant hormone SA is well known as an endogenous signal in plant defense responses (Shah, 2003; Loake and Grant, 2007); however, there is evidence supporting its role in plant growth and developmental processes via regulating cell division and expansion (Li et al., 2022). Therefore, the observation of markedly and moderately high level of endospermic SA in both tissues during the early and late stages of SF of barley seed development, respectively, might reflect its importance in the control of cell division and expansion in the embryo and endosperm of developing barley seeds tissues. The prevalence of close associations between endospermic SA level and the expression patterns of *HvPAL5* and *HvSGT* during SF reflects the role of both SA biosynthesis and inactivation in controlling SA level in the endosperm of developing barley seeds. Given previous studies reported that SA regulates PCD in response to abiotic stress (Bernacki et al., 2021), the presence of elevated endospermic SA level during post-PM phase might indicate its role in regulating programmed death of endospermic cells during barley seed maturation. Our results also showed that SA level during post-PM is mediated mainly by the expression of *HvICS*.

Developing barley seeds attain highest level of dormancy at their PM, which occurs 25 and 30 DAA (Sreenivasulu et al., 2006; Bewley et al., 2013). Consistently, the level of embryonic ABA increased during SF and peaked at PM. In addition, much higher level of ABA was detected in the embryo than endosperm during the same period. The increase in embryonic ABA level during SF through PM is associated with the expression patterns of the two *HvNCED* genes. Previous studies have also shown the occurrence of a peak in embryonic ABA level during physiological maturity of barley seeds and its regulation via expression of *HvNCED2* (Benech-Arnold et al., 1999; Chono et al., 2006). However, the difference in ABA level between the embryo and endosperm tissues is not associated with the expression patterns of either ABA biosynthesis or catabolic genes. Although embryonic ABA plays important roles in dormancy induction and maintenance during seed maturation in barley (Benech-Arnold et al., 1999), several reports implicated the balance between the levels of ABA and GA as the main factor controlling these seed developmental events (Tuan et al., 2018a; Tuan et al., 2018b). In agreement with these reports, the relatively higher levels of embryonic bioactive GAs detected at early stage of SF declined to undetectable level by PM, leading to the prevalence high ratio of ABA level to GA levels. In agreement with these results, ABA and GA regulated transcriptional networks have been reported to have critical roles in the control of dormancy acquisition and maintenance during maturation of wheat seeds (Yamasaki et al., 2017). The decrease in the levels of embryonic GAs during the same period is associated with the expression patterns of *HvGA20ox*s and *HvGA3ox*s, and *HvGA2ox6*, reflecting the importance of both GA biosynthesis and catabolism in regulating embryonic GA level and thereby establishment of dormancy during barley seed development. The prevalence of a relatively lower amount of embryonic ABA during post-PM phase, when no bioactive GA was detected, as compared to that observed at PM might underlie the weak dormancy manifested in the seeds of cv. Morex at harvest maturity (Rasmusson and Wilcoxson, 1979; Han et al., 1996). Our study also indicated that the level ABA in the embryo during post-PM is associated with the expression patterns of *HvNCED*s and *HvCYP707A1*.

Other hormones such as auxin, JA, CK and SA are also involved in the regulation of seed dormancy; however, their role in the induction and maintenance of dormancy during seed maturation in barley is yet to be elucidated. Previous reports implicated auxin as positive regulator of seed dormancy in wheat (Morris et al., 1988; Ramaih et al., 2003). Thus, the decline in embryonic IAA level during barley seed maturation, might imply the minimal role of auxin in the establishment and retention of seed dormancy. In agreement with our result, a decrease in embryonic IAA level has been observed during maturation of wheat seeds irrespective of variation in their level of dormancy (Tuan et al., 2019). The association of embryonic IAA level with the expression patterns of *HvTAA1*, *HvYUC4* and *HvYUC5* reflects the role of IAA biosynthesis rather than its inactivation in controlling IAA level in the embryo of developing barley seeds. It has been shown previously that jasmonate induces seed dormancy decay in seeds of cereal species such as wheat (Jacobsen et al., 2013; Nguyen et al., 2022). Consistent with these reports, dormancy loss in barley seeds due to after-ripening is associated with enhanced expression levels of specific JA biosynthesis and signaling genes (Barrero et al., 2009). Given cv. Morex of barley produces seeds with low level of dormancy at harvest maturity (Rasmusson and Wilcoxson, 1979; Han et al., 1996), the prevalence of low level of embryonic JA-Ile during PM and post-PM might indicate that the induction and maintenance of dormancy during maturation of barley seeds is not JA dependent. Our data also revealed that embryonic JA-Ile level during the same period is regulated via expression of the JA biosynthesis genes studied.

Cytokinins regulate seed dormancy negatively (Kucera et al., 2005); therefore, the detection of low level of embryonic IPA, *t*Z and dZ in maturing seeds of cv. Morex suggests the minimal role of CK in regulating of seed dormancy during barley seed maturation. In contrast, a previous study in wheat reported the presence of higher levels of *t*Z during maturation of wheat seeds that exhibit low level of dormancy at maturity as compared to those exhibiting high level of dormancy (Tuan et al., 2019). Therefore, further study is required to elucidate the role of CKs in regulating dormancy in barley and other cereal seeds. The prevalence of close associations between embryonic CK levels and expression patterns of *HvLOG3* and *HvZOG1* reflects the importance of both CK biosynthesis and inactivation in the control of CK levels in the embryo of maturing barley seeds. It has been shown previously that exogenous SA inhibits barley seed germination and GA-induced activity of α-amylase in the aleurone cells via ABA-inducible *WRKY* gene (Xie et al., 2007). Thus, the reduction of embryonic SA level as the seeds transitioned to PM and during post-PM might indicate its contribution to the low level of dormancy manifested in the mature seeds of cv. Morex. The observed reduction of embryonic SA level during the same seed developmental period is closely associated with the expression patterns of *HvPAL5* and *HvSGT*, implying the importance of both SA biosynthesis and inactivation in regulating SA level in the embryo of maturing barley seeds.

**Figure 9:**
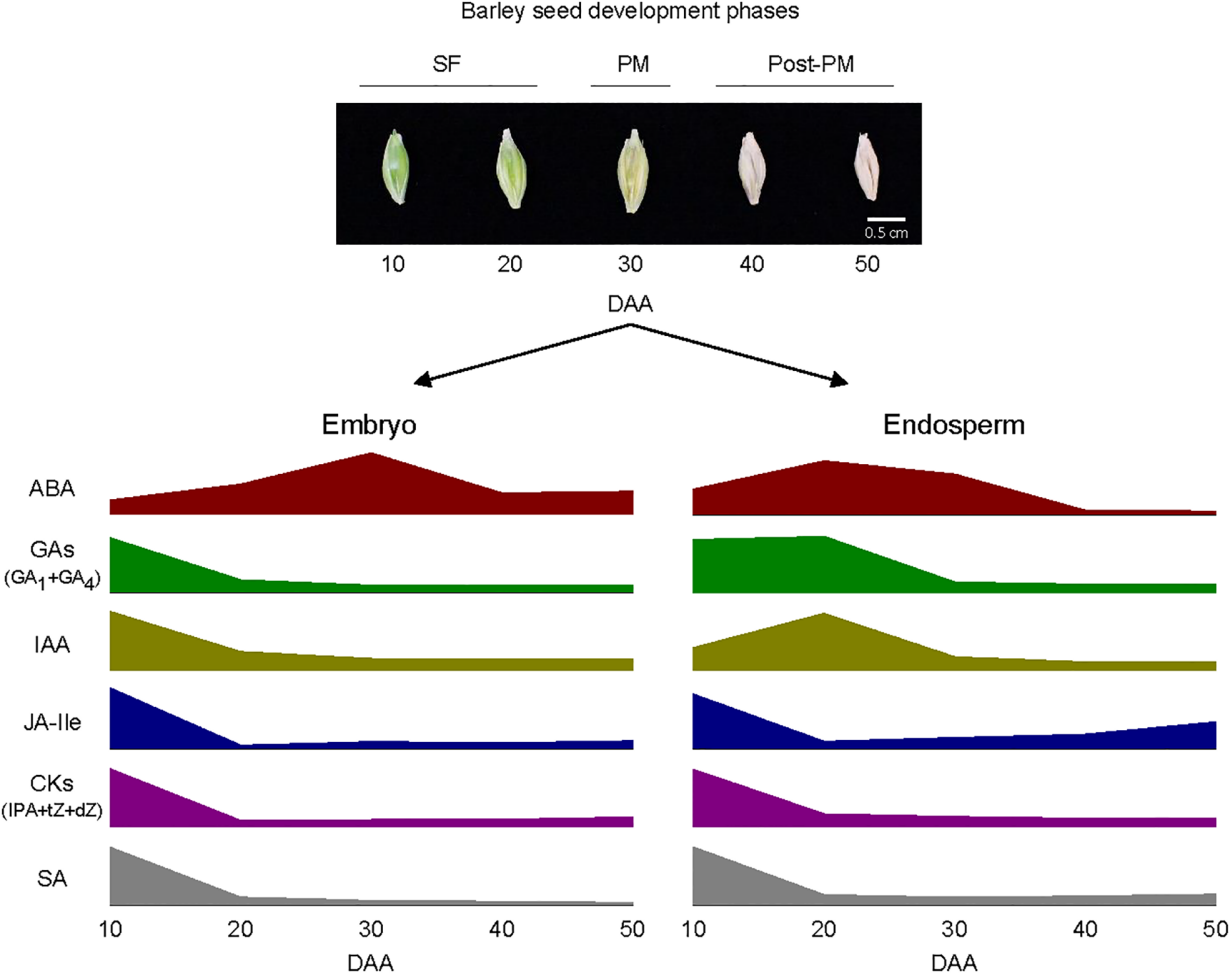
Schematic diagram depicting the pattern of changes in the levels of bioactive forms of plant hormones within embryo and endosperm tissues during barley seed development. Elevated levels of abscisic acid (ABA) and relatively high levels of gibberellins (GAs; GA_1_ and GA_4_), indole acetic acid (IAA), jasmonate iso-leucine (JA-Ile), cytokinins [CKs; isopentyladenine (IPA), *trans*-zeatin (*t*Z) and dihydrozeatin (dZ)] and salicylic acid (SA) characterize both embryo and endosperm tissues during the early and/or late stage of seed filling (SF). The embryo exhibit a peak in ABA level around physiological maturity (PM) and the endosperm also consist of a relatively high level of ABA at this stage; both tissues exhibit relatively lower level of ABA during post-PM phase. Both embryo and endosperm tissues contain low levels of all the other plant hormones at PM and during post-PM phases except that the endosperm exhibit relatively higher levels of JA-Ile and SA at the late stage of post-PM phase. See **Figure 3** for variations in the levels of the plant hormones between the two tissues as such variation is not depicted in the diagram.

In summary, this study showed the temporal and spatial modulation of the levels of bioactive forms of plant hormones during seed development in barley as depicted in **Figure 9**, and this modulation of plant hormone levels is mediated by transcriptional regulation of genes involved in their respective metabolic pathways. These results provide important insights into potential roles of phytohormones in the regulation of seed developmental processes such as SF as well as induction and maintenance of dormancy in barley, important agronomic traits that play significant roles in determining its yield and quality. Given the roles of plant hormones in seed development are also mediated by their distribution and tissue sensitivity, elucidating the transcriptional regulation of their transport and signal transduction is critical to extend our understanding of hormonal regulation of seed development in barley.

## Data availability statement

The original contributions presented in the study are included in the article/**Supplementary Material**. Further inquiries can be directed to the corresponding author.

## Author contributions

BA conceived the research; BA and PA designed the experiments; PA and PT performed the experiments; PT and T-NN analyzed the data and wrote the manuscript; BA reviewed and edited the manuscript. All authors contributed to the article and approved the submitted version.
